# Characterizing skin and soft tissue infections in patients with cancer on systemic oncologic therapy: A single institution retrospective analysis from the outpatient and inpatient oncodermatology service

**DOI:** 10.1016/j.jdin.2023.11.011

**Published:** 2023-12-29

**Authors:** Jolanta J. Pach, Caroline A. Nelson, Jonathan S. Leventhal

**Affiliations:** Department of Dermatology, Yale School of Medicine, New Haven, Connecticut

**Keywords:** cancer therapy, dermatologic toxicity, dermatology, oncodermatology, oncology, skin infection, soft tissue infection

*To the Editor:*Skin and soft tissue infections (SSTIs) are common complications of systemic cancer therapy.^1^^,^^2^ Immunosuppression from malignancy or oncologic treatment may also predispose patients to SSTIs.^1^^,^^3^^,^^4^ Studies investigating infections associated with various types of medical oncologic therapy are lacking.

We aimed to characterize systemic cancer therapy-related SSTIs in the outpatient and inpatient consultative services by means of a retrospective chart review of the Yale New Haven Health electronic medical record from 2016 to 2022. Patients actively treated with cytotoxic chemotherapy, targeted therapy, and/or immunotherapy were included. Infections were identified by search of electronic medical record using keywords “infection,” “cellulitis,” “abscess,” “bacterial,” “fungal,” “viral,” “parasitic,” and related terms and confirmed by manual review of dermatology notes.

There were 239 (136 outpatient and 103 inpatient) SSTIs among 209 patients (Table I). The majority occurred in patients receiving regimens including cytotoxic therapy [alone or with concurrent targeted agents and/or immunotherapy (67.4%)]. In comparison, fewer infections were reported with targeted agents [alone or with concurrent immunotherapy (27.2%)] and immunotherapy alone (5.4%).

**Table I:** Characteristics of cancer patients on systemic oncologic therapy in our cohort

Characteristic	Total (*n* = 209)	Outpatient (*n* = 124)	Inpatient(*n* = 85)
Mean age	62.4	63.9	60.4
Sex			
Male	108	66	42
Female	101	58	43
Race∗			
White/Caucasian	165	102	63
Black/African American	21	9	12
Asian	6	3	3
American Indian or Alaska Native	1	1	
Native Hawaiian or other Pacific Islander	1		1
Other	13	7	6
Ethnicity∗			
Non-Hispanic	189	113	76
Hispanic	18	10	8
Cancer diagnosis			
Hematologic cancer	88	36	52
Lung cancer	25	20	5
Breast cancer	24	18	6
Gastrointestinal cancer	17	10	7
Genitourinary cancer	12	10	2
Head and neck squamous cell cancer	12	11	1
Gynecologic cancer	10	5	5
Skin cancer	10	6	4
Brain cancer	7	5	2
Other	4	3	1

∗ Two patients refused.

Most infections were bacterial (*n* = 112, 46.9%), including superficial and deep infections. Others included 69 fungal (28.9%), 53 viral (22.2%), 4 ectoparasitic (1.7%), and 1 atypical mycobacterial (0.4%) infections (Table II). Superficial bacterial infections were frequently associated with targeted therapy (eg, secondarily infected acneiform eruption) (Supplementary Table I, available via Mendeley at https://data.mendeley.com/datasets/44sv53vtrk/1) whereas cytotoxic therapy was commonly associated with invasive or disseminated infections: 60.8% of bacterial infections involved the soft tissues, 6.8% of fungal infections were invasive, and 17.9% of viral infections were disseminated. Atypical infections (eg, mucormycosis, nocardiosis, mycobacterium) predominantly occurred with cytotoxic therapy and hematologic malignancy. Overall, 63.0% of invasive or disseminated infections were associated with hematologic malignancy.

**Table II:** Skin and soft tissue infections in patients with cancer on systemic oncologic therapy

Associated systemic oncologic therapy	Bacterial		Atypical mycobacterial	Fungal		Viral		Ectoparasitic
	Superficial infection∗	Soft tissue infection†	Soft tissueinfection	Superficial infection‡	Soft tissue infection§	Localized infection‖	Disseminated infection¶	Superficial infection#
Cytotoxic therapy∗∗ (*n* = 161)	29 (39.2%)	45 (60.8%)		41 (93.2%)	3 (6.8%)	32 (82.1%)	7 (17.9%)	4 (100.0%)
Targeted therapy†† (*n* = 65)	21 (60.0%)	14 (40.0%)	1 (100.0%)	19 (100.0%)		9 (90.0%)	1 (10.0%)	
ICIs (*n* = 13)	1 (33.3%)	2 (66.7%)		6 (100.0%)		4 (100%)		

Cytotoxic therapy regimens were most often antimetabolite (23.6%), combined antimetabolite and alkylating (19.9%), combined antimicrotubule and alkylating (12.4%), and alkylating (11.8%) agents. Targeted therapies most often included medications that block epidermal growth factor receptor (17.6%), vascular endothelial growth factor receptor (11.5%), B cell antigen CD20 (9.9%), and human epidermal growth factor receptor 2 (7.6%). Immunotherapy primarily included antiprogrammed cell death protein 1 (71.4%) and combined antiprogrammed cell death protein 1 and anti-cytotoxic T-lymphocyte antigen 4 (17.1%) therapy.

*ICI*s, Immune checkpoint inhibitors.

∗ Diagnoses include superficial folliculitis, impetigo, bacterial intertrigo, and bacterial paronychia.

† Diagnoses include cellulitis, erysipelas, abscess, furunculosis, ecthyma, ecthyma gangrenosum, and cutaneous nocardiosis.

‡ Diagnoses include dermatophytosis (tinea capitis, corporis, cruris, and pedis, onychomycosis, and Majocchi granuloma), mucocutaneous candidiasis, and pityrosporum folliculitis.

§ Diagnoses include invasive candidiasis (candidal abscesses with candidemia) and mucormycosis.

‖ Diagnoses include herpes simplex virus, zoster, and perianal condyloma.

¶ Diagnoses includes disseminated herpes simplex virus, disseminated or multidermatomal zoster, varicella, and coxsackievirus.

# Diagnoses include scabies and demodex folliculitis.

∗∗ Includes infections associated with cytotoxic therapy alone (*n* = 84, 35.1%) and with concurrent targeted therapy (*n* = 48, 20.1%), history of hematopoietic stem cell transplant (*n* = 20, 8.4%), concurrent ICI therapy (*n* = 5, 2.1%), and concurrent targeted plus ICI therapy (*n* = 4, 1.7%).

†† Includes infections associated with targeted therapy alone (*n* = 52, 21.8%) and with concurrent ICI therapy (*n* = 13, 5.4%).

One hundred seventy cases (71.1%) (88 [64.7%] outpatient, 82 [79.6%] inpatient) received systemic antimicrobials, with 146 (61.1%) and 51 (21.3%) receiving oral and intravenous antimicrobials, respectively; 132 (55.2%) (97 [71.3%] outpatient, 35 [34.0%] inpatient) received topical antimicrobials, while 3 patients (1.3%) (3 [2.9%] inpatient) only received supportive care or refused antimicrobial treatment. Invasive or disseminated infections often received inpatient treatment (63.0%). Seven patients (2.9%) had cancer therapy interruption (5 [2.1%]) or cessation (2 [0.8%]) due to concern for primary SSTIs, the majority of which were invasive or disseminated (85.7%) rather than superficial (14.3%).

Limitations of our study include its retrospective nature and data from a single institution. Diagnoses were based on clinicopathological correlation supported by superficial wound culture and/or sterile tissue culture results as available. In the absence of data on the number of patients receiving different treatment modalities or concurrent immunosuppressive agents unrelated to malignancy treatment at our institution during the study period, caution is required when drawing conclusions as to which treatment modalities are most associated with infections. Nevertheless, in our study population, invasive bacterial infections, disseminated viral infections, invasive fungal infections, and atypical infections occurred primarily in patients treated with regimens including cytotoxic therapy or with history of hematologic malignancy. Invasive or disseminated infections were also more likely to require inpatient management or impact cancer therapy. Therefore, the threshold for obtaining sterile tissue cultures for bacterial, fungal, and mycobacterial organisms should be low in these patients to avoid life-threatening complications.

This study characterizes the varied presentations of SSTIs in patients on systemic cancer therapy from a tertiary care center; multiinstitutional prospective studies are needed to further investigate our findings.

## Conflicts of interest

Dr Leventhal serves on the advisory boards of La Roche-Posay and Sanofi and Regeneron Pharmaceuticals. Dr Nelson received an unrelated grant from Boehringer Ingelheim. Author Pach has no conflicts of interest to declare.

## References

[1] Gandhi M., Brieva J.C., Lacouture M.E. (2014). Dermatologic infections in cancer patients. Cancer Treat Res.

[2] Phillips G.S., Freites-Martinez A., Hsu M. (2018). Inflammatory dermatoses, infections, and drug eruptions are the most common skin conditions in hospitalized cancer patients. J Am Acad Dermatol.

[3] Hirotsu K., Dang T.M., Li S. (2019). Association of antibiotic resistance with antibiotic use for epidermal growth factor receptor inhibitor-related papulopustular eruption. JAMA Dermatol.

[4] Ferreira M.N., Ramseier J.Y., Leventhal J.S. (2019). Dermatologic conditions in women receiving systemic cancer therapy. Int J Womens Dermatol.

